# A Comparison of the Cecal Microbiota between the Infection and Recovery Periods in Chickens with Different Susceptibilities to *Eimeria tenella*

**DOI:** 10.3390/ani14182709

**Published:** 2024-09-18

**Authors:** Jianqiang Tang, Qi Wang, Hailiang Yu, Liyue Dong, Meihui Tang, Areej Arif, Genxi Zhang, Tao Zhang, Kaizhou Xie, Shijie Su, Zhenhua Zhao, Guojun Dai

**Affiliations:** 1 College of Animal Science and Technology, Yangzhou University, Yangzhou 225000, China; 2 Poultry Institute, Chinese Academy of Agricultural Sciences, Yangzhou 225125, China

**Keywords:** *E. tenella*, cecal microbiota, resistance selection, metagenome sequencing

## Abstract

**Simple Summary:**

Coccidiosis is an intestinal parasitic disease caused by *Eimeria* protozoa, which endangers the health and growth of animals. However, little is known about the intestinal resident microbiota of chickens with different resistance to *Eimeria tenella* (*E. tenella*) infection and the changes in the microbiota from infection to recovery periods. The metagenomic sequencing analyses show that many potential differential microbial taxa were identified in different resistant chickens, respectively. Through the comparative analysis from infection to recovery periods, the number of microorganisms with the same trend of change between the control and resistant groups was greater than that between the control and susceptible groups. These findings provide a reference for further research into how *E. tenella* infection affects the intestinal microbiota and thus the growth, development, and health of chickens.

**Abstract:**

To investigate the effect of *Eimeria tenella* (*E. tenella*) infection on the cecal microbiota, resistant and susceptible families were screened out based on the coccidiosis resistance evaluation indexes after *E. tenella* infection. Subsequently, a comparative analysis of cecal microorganisms among control, resistant, and susceptible groups as well as between different periods following the *E. tenella* challenge was conducted using metagenomic sequencing technology. The results showed that the abundance of opportunistic pathogens, such as *Pantoea*, *Sporomusa*, and *Pasteurella* in the susceptible group and *Helicobacter* and *Sutterella* in the resistant group, was significantly higher on day 27 post-inoculation (PI) (the recovery period) than on day 5 PI (the infection period). Additionally, the abundance of *Alistipes*, *Butyricicoccus*, and *Eubacterium* in the susceptible group and *Coprococcus*, *Roseburia*, *Butyricicoccus*, and *Lactobacillus* in the resistant group showed a significant upward trend during the infection period compared with that in the recovery period. On day 5 PI, the abundance of *Faecalibacterium* and *Lactobacillus* was decreased in both the resistant and susceptible groups when compared with that in the control group and was greater in the resistant group than in the susceptible group, while *Alistipes* in the susceptible group had a relatively higher abundance than that in other groups. A total of 49 biomarker taxa were identified using the linear discriminant analysis (LDA) effect size (LEfSe) method. Of these, the relative abundance of *Lactobacillus aviarius*, *Lactobacillus salivarius*, *Roseburia*, and *Ruminococcus gauvreauii* was increased in the resistant group, while *Bacteroides_sp__AGMB03916*, *Fusobacterium_mortiferum*, *Alistipes_sp__An31A*, and *Alistipes_sp__Marseille_P5061* were enriched in the susceptible group. On day 27 PI, LDA scores identified 43 biomarkers, among which the relative abundance of *Elusimicrobium_sp__An273* and *Desulfovibrio_sp__An276* was increased in the resistant group, while that of *Bacteroides_sp__43_108*, *Chlamydiia*, *Chlamydiales*, and *Sutterella_sp__AM11 39* was augmented in the susceptible group. Our results indicated that *E. tenella* infection affects the structure of the cecal microbiota during both the challenge and recovery periods. These findings will enhance the understanding of the effects of changes in the cecal microbiota on chickens after coccidia infection and provide a reference for further research on the mechanisms underlying how the intestinal microbiota influence the growth and health of chickens.

## 1. Introduction

Coccidiosis is a common parasitic disease in poultry farming. Affected animals display poor nutrient absorption, lower feed conversion rates, reduced body weight gain, and decreased productivity, finally resulting in significant economic losses [1,2,3]. *Eimeria tenella* (*E. tenella*) is one of the most pathogenic species in the *Eimeria* genus. This coccidia mainly parasitizes the cecum and causes an imbalance in the intestinal environment by specifically invading and damaging the epithelial cells of the cecum and disrupting the composition of the cecal microbiota [4,5].

The cecal microbiota of poultry is highly diverse and plays a key role in host nutrient absorption [6]. Beneficial microorganisms maintain the intestinal health of their hosts by promoting nutrient absorption and energy acquisition, thus stimulating the intestinal immune system and helping to defend against invasion by harmful microbes [7]. For example, *Faecalibacterium* is involved in the absorption of sugars in the intestine, promotes the production of short-chain fatty acids (SCFAs), and provides the host with essential amino acids and butyrate, which is beneficial in reducing the severity of *E. tenella* infection in poultry [8,9]. Studies have shown that *E. tenella* infection in chickens promotes the proliferation of pathogenic bacteria such as *Clostridium perfringens* (causes necrotizing enterocolitis), *Salmonella enteritidis*, and *Campylobacter jejuni*, as well as colonization by other pathogens, thus increasing the risk of other diseases and greatly increasing mortality [10].

*Lactobacillus* stimulates the production of immune cytokines such as interleukin (IL)-1β, IL-6, and interferon-gamma (IFN-γ) in the intestine and contributes to relieving intestinal inflammation caused by *E. tenella* infection [11,12]. Swaggerty et al. [13] showed that chickens with higher inherent levels of pro-inflammatory mediators have enhanced innate immunity, which mitigates the pathological damage and loss of productivity resulting from coccidial infection. Macdonald et al. [14] showed that chickens with different cecal lesion damage scores after *E. tenella* infection had significantly distinct intestinal microbiota abundance, characterized by an increase in the prevalence of *Enterobacteriaceae* and a decrease in that of *Bacillales* and *Lactobacillus*. In recent years, gut microbiota-based interventions have been considered to be effective in treating coccidiosis [15,16]. However, little is known about the effects of *E. tenella* infection on the intestinal microbiota among chickens with different levels of resistance, as well as the changes in the resident intestinal microbiota of chickens from infection to recovery periods.

In this study, employing metagenomic sequencing, we compared the changes in cecal microbiota composition and abundance among chickens with different levels of resistance to *E. tenella* following challenge with the parasite and explored the changes in cecal microbiota between the infection and recovery periods. These results provide a reference for further research into how *E. tenella* infection affects the intestinal microbiota and thus the growth, development, and health of chickens.

## 2. Materials and Methods

### 2.1. Ethics Approval

The animal experiment protocols were approved by the Animal Welfare Committee of Yangzhou University [permit number: SYXK (Su) IACUC 2012-0029].

### 2.2. Oocysts

*E. tenella* of the Yangzhou strain were provided by the Parasitology Department of the College of Veterinary Medicine, and the oocysts were propagated in 3–4-week-old chickens. Oocysts were isolated and purified from feces by salt flotation and centrifugation, sporulated in vitro at 28 °C, and stored in a 2.5% potassium dichromate (K_2_Cr_2_O_7_) solution at 4 °C.

### 2.3. Bird Husbandry and Experimental Design

Seven half-sib Jinhai yellow chicken families (A–G) were generated from full-sibling parents through artificial insemination. All chickens were housed in pathogen-free isolation cages hanging off the floor by flame sterilization (one cage, one chicken) at the temperature of 20~28 °C and humidity of 50~65%. All chickens were not vaccinated against coccidia and provided with complete feed and clean water without anticoccidial drugs.

Each family (*n* = 32) was randomly divided into two groups—a control group (CG, *n* = 12) and an infection group (IG, *n* = 20) at the age of 30 days. Each bird in the IG groups was orally challenged with 3.5 × 10^4^ sporulated *E. tenella* oocysts (water was not provided within 6 h after challenge to ensure that the oocysts were ingested by the birds), while birds in the CG groups were given the same amount of normal saline.

On day 5 post-inoculation (PI), 10 chickens of each IG were randomly selected and euthanized by rapid cervical dislocation for coccidiosis resistance evaluation. After assessment, the most resistant and susceptible families were selected from the seven families. Then the IG of the most resistant and susceptible families were named as resistant (JR) and susceptible (JS) groups, respectively, while the CG of the corresponding 2 families was combined as a new control group (JC) for subsequent studies. Meanwhile, the cecum contents of 4 female chicks out of 10 chickens were randomly collected from resistant, susceptible, and control groups. The remaining chickens of each group were fed until day 27 PI, and the cecum contents were sampled using the same method as on day 5 PI. The cecum contents of chickens were stored in liquid nitrogen in sterile tubes for cecal microbial metagenomic sequencing.

### 2.4. Assessment of Coccidiosis Resistance

Coccidiosis resistance was assessed based on the following indexes, including relative weight gain (RWG), cecal lesion scores, oocysts per gram (OPG), and anticoccidial index (ACI). The weight gain in each group was calculated from day 0 to day 5 PI. The RWG was calculated as (average weight gain in the IG/average weight gain in the CG) × 100%. On day 5 PI, the cecal lesion scores of 10 chickens selected from each IG were obtained according to the international 4-point cecum lesion scoring standard [17]. The number of OPG was measured in feces, evenly sampled from five points on the cage floor on day 5 PI [18]. The ACI was calculated using the following formula: ACI =  (survival rate + relative weight gain)—(lesion sore + oocyst index) according to McManus et al. [19,20]. The anticoccidial effect of chickens was considered excellent when ACI ≥ 180, good when 160 ≤ ACI < 180, fair when 120 ≤ ACI < 160, and no anticoccidial effect when ACI < 120.

### 2.5. DNA Extraction and Metagenome Sequencing Analysis

DNA was isolated from cecal microbial genomic DNA using the MagPure Soil DNA KF Kit (Hybribio, Hong Kong, China) following the manufacturer’s instructions. DNA concentration and integrity were assessed by a NanoDrop2000 spectrophotometer (Thermo Fisher Scientific, Waltham, MA, USA) and agarose gel electrophoresis, respectively. Library construction was performed using the TruSeq Nano DNA LT Sample Preparation Kit (Illumina, San Diego, CA, USA). Libraries were sequenced using the Illumina NovaSeq 6000 sequencing platform, and 150-bp paired-end reads were generated (the sequencing volume is 10 G). Library construction and sequencing were carried out by OE Biomedical Technology Co., Ltd. (Shanghai, China).

After sequencing, reads were trimmed and filtered using Trimmomatic (v.0.36). The post-filtered pair-end reads were aligned against the host genome using bowtie2 (v2.2.9), and the aligned reads were discarded. Metagenome assembly was performed using MEGAHIT (v1.1.2) after obtaining valid reads. ORF prediction of assembled scaffolds using prodigal (v2.6.3) was performed and translated into amino acid sequences. The non-redundant gene sets were built for all predicted genes using CDHIT (v4.6.7), and clustering parameters were 95% identity and 90% coverage. The longest gene was selected as the representative sequence of each gene set. The clean reads of each sample were aligned against the non-redundant gene set (95% identity) using bowtie2 (v.2.2.9), and gene abundance information in the corresponding sample was counted.

Species classification was obtained from the corresponding taxonomy database in the NR Library, while species abundance was calculated using the relative expression level of the corresponding genes. An abundance profile was generated at the levels of domain, kingdom, phylum, class, order, family, genus, and species. Alpha-diversity analysis and the plotting of the species abundance spectrum or the functional abundance spectrum were carried out using R software (v.3.2.0). To obtain beta diversity, the distance matrices were calculated using the Bray Curtis distance algorithm and visualized by the principal coordinate analysis (PCoA) using R (v 3.2.0), followed by the PERMANOVA statistical test (*p* < 0.05). The linear discriminant analysis (LDA) effect size (LEfSe) algorithm was used to calculate the cecal microbial LDA scores and screen out the differential cecal microbiota using *p* < 0.05 and an LDA effect value (score) of 2 as the selection criteria. The Fruchterman–Reingold layout algorithm was used to generate a plot of the microbiome colony network via the interactive platform Gephi (v.9.2); correlations with |*r*| > 0.8 and *p* < 0.05 were regarded as statistically significant and were used to build a co-occurrence network.

### 2.6. Statistical Analysis

Body weight differences between CG and IG were tested by an independent-samples T test. Differences in the coccidiosis resistance evaluation indexes were analyzed by a Kruskal–Wallis test in SPSS 25.0 software (SPSS Inc., Chicago, IL, USA). *p* < 0.05 indicated significant differences, while *p* < 0.01 indicated highly significant differences. To distinguish the temporal effect and the treatment effect, the Scheirer–Ray–Hare test was used for a two-way factorial analysis at the genus level; *p* < 0.01 indicated highly significant differences. The data are presented as means ± SD.

## 3. Results

### 3.1. Construction of E. tenella-Resistant and E. tenella-Susceptible Chickens

As shown in Table 1, there was no significant difference in initial weight (before *E. tenella* infection) between the CG and IG (*p* > 0.05). However, there was a highly significant difference in final body weight between the CG and IG in families A, B, and C (*p* < 0.01); there was also a significant difference between the CG and IG in families D and E (*p* < 0.05). Family D had the highest RWG (61.47%), and family C had the lowest (16.62%), a difference of 44.85%. This indicated that family D was the most resistant and family C the most susceptible to E. tenella infection.

**Figure 1:**
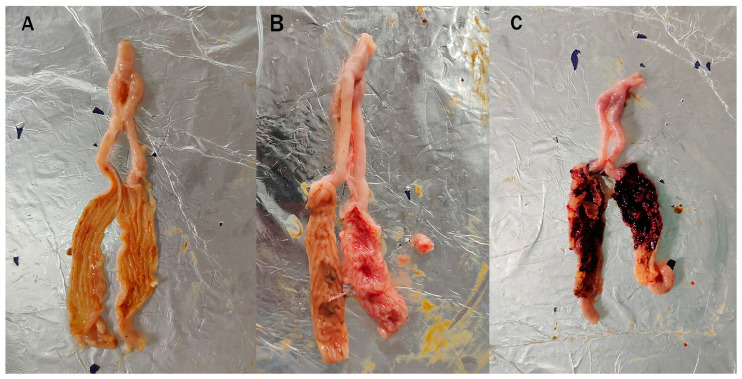
Images of cecal tissues of chickens on day 5 after *E. tenella* infection: (**A**) control group; (**B**) resistant group in family D; (**C**) susceptible group in family C.

As shown in Table 2, the number of OPG of feces discharged in family C was 12.42 × 10^6^ /g, which was significantly higher than that in all the other families (*p* < 0.01); meanwhile, family D had the lowest OPG among the seven families. Regarding the cecal lesion score, family C had the highest score, and family D had the lowest. The ACI of family C was 46.62, which was significantly lower than that in all the other families (*p* < 0.01), while the ACI of family D reached 151.47, which was significantly higher than that in all the other families (*p* < 0.01). These results further supported that family C was the most susceptible to coccidia, whereas family D was the most resistant. Then the IG of family C and D were named as susceptible (JS) and resistant (JR) groups, respectively, while the CG of family C and D were mixed into the control group (JC). In addition, compared with birds in the control group (Figure 1A), the surface of the cecal mucosa of chickens in the resistant group of family D had fewer hemorrhagic spots, and the cecal wall was only slightly swollen and thickened (Figure 1B); in contrast, the cecal wall of chickens in the susceptible group in family C was extremely thickened and the mucosa was covered with hemorrhagic patches (Figure 1C). Combined, these findings indicated that a model of *E. tenella* infection-resistant and *E. tenella* infection-susceptible chickens had been successfully constructed.

### 3.2. Diversity of the Cecal Microbiota

**Figure 2:**
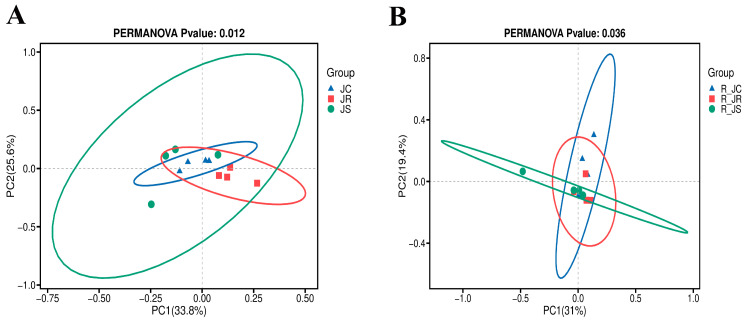
Composition comparison of cecal microbiota at the species level among the JC, JR, and JS groups during the infection and recovery periods: (**A**) the infection period; (**B**) the recovery period. PCoA plots of beta diversity are based on the Bray–Curtis distances followed by the PERMANOVA statistical test. JC, control group; JR, resistant group; JS, susceptible group; R_JC, control group in the recovery period; R_JR, resistant group in the recovery period; R_JS, susceptible group in the recovery period; PCoA, principal coordinate analysis.

We investigated the richness and diversity (alpha diversity) of microbial species using the Ace index, Shannon index, and Simpson index, respectively. As shown in Table 3, although there was no significant difference in cecal microbiota alpha diversity among the control, resistant, and susceptible groups during the infection and recovery periods (*p* > 0.05), with *E. tenella* recovery, the microbiota richness of the three groups decreased in the recovery period than in the infection period, while the microbiota overall diversity of the three groups increased in the recovery period than in the infection period. As shown in Figure 2, the dispersion of distances between samples from different resistance groups during the infection period was more pronounced at the species level, and the composition of the cecum microbial community differed significantly between each group (*p* < 0.05) (Figure 2A). During the recovery period, the plot of PcoA exhibited a tendency toward change, and the samples exhibited partial similarity between the three groups, with a large regional overlap, and PERMANOVA analysis showed that the composition of the cecum microbial community still differed significantly (*p* < 0.05) between the different resistance groups (Figure 2B).

### 3.3. Changes in the Cecal Microbiota after E. tenella Infection

#### 3.3.1. Comparison of the Cecal Microbiota from the Infection Period to the Recovery Period at the Phylum Level

**Figure 3:**
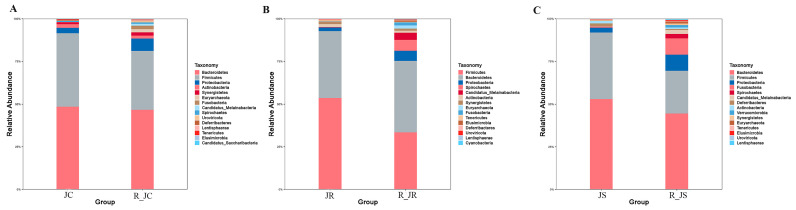
Comparison of the relative abundance of the top 15 most abundant cecal microorganisms at the phylum level in the different experimental periods: (**A**) JC vs. R_JC group; (**B**) JR vs. R_JR group; (**C**) JS vs. R_JS group. JC, control group; JR, resistant group; JS, susceptible group; R_JC, control group in the recovery period; R_JR, resistant group in the recovery period; R_JS, susceptible group in the recovery period.

The comparison of the top 15 most abundant microorganisms in the cecal microbiome at the phylum level for each group between the infection and recovery periods is shown in Figure 3. The three most prevalent phyla in the cecum microflora in all the groups were *Bacteroidetes*, *Firmicutes*, and *Proteobacteria*. The abundance of *Firmicutes* in the control group showed a decreasing trend from the infection period to the recovery period, while the *Proteobacteria* showed an increasing trend (differences of 8.62 and −4.09, respectively) (Figure 3A). For the resistant group, the abundance of *Firmicutes* showed a decreasing trend between the two periods (a difference of 20.1), whereas *Spirochaetes* showed an increasing trend, with a difference of −6.26 (Figure 3B). For the susceptible group, meanwhile, *Firmicutes* showed a decreasing trend, with a difference of 13.90 (Figure 3C). Notably, the changes in the abundance of the dominant phyla, including *Bacteroidetes*, *Firmicutes*, and *Proteobacteria*, were greater in the infection group than in the control group both in the *E. tenella* infection period and the recovery period.

#### 3.3.2. Differential Abundance Analysis of the Cecal Microbiota from the Infection Period to the Recovery Period at the Genus Level

**Figure 4:**
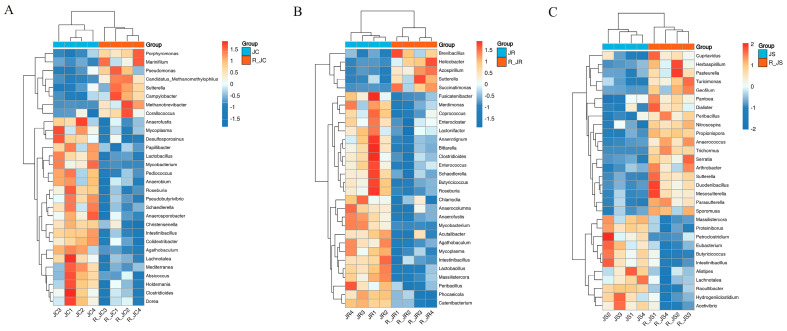
Comparative analysis of the differential abundance of cecal microbiota between the infection period and the recovery period in each group. Hierarchical clustering plots of the top 30 microorganisms with significant differences in abundance at the genus level: (**A**) JC vs. R_JC group; (**B**) JR vs. R_JR group; (**C**) JS vs. R_JS group. The bar represents the maximum and minimum values of a set of data. Statistical analysis was performed using the Wilcoxon rank−sum test (*p* < 0.05). JC, control group; JR, resistant group JS, susceptible group; R_JC, control group in the recovery period; R_JR, resistant group in the recovery period; R_JS, susceptible group in the recovery period.

The Scheirer–Ray–Hare test has shown that, at the genus level, there were 529 cecal microorganisms with highly significant differences between the infection and recovery periods (*p* < 0.01). Then we used the Wilcoxon rank-sum test to analyze the differential abundance of cecal microbiota in each treatment from infection to recovery periods. The top 30 cecal microorganisms with significant differences were shown in Figure 4. In the control group, we found that 8 cecal microbiotas significantly increased from the infection period to the recovery period, while 22 significantly decreased (*p* < 0.05, Figure 4A). In the resistant group, only 5 cecal microbiotas significantly increased from the infection period to the recovery period, such as *Helicobacter* and *Sutterella*, while 25 decreased significantly, such as *Coprococcus*, *Roseburia*, *Butyricicoccus*, and *Lactobacillus* (*p* < 0.05, Figure 4B). In the susceptible group, it is noteworthy that there were 19 cecal microbiotas that significantly increased in the recovery period, such as *Pantoea*, *Sporomusa*, and *Pasteurella*, while only 11 cecal microbiotas decreased significantly in the recovery period, such as *Alistipes*, *Butyricicoccus*, and *Eubacterium* (*p* < 0.05, Figure 4C). In addition, we also found that 10 cecal microbiotas had the same change trend from infection to recovery periods in the control and resistant groups, while only 3 cecal microbiotas had the same trend in the susceptible and control groups. Thus, the cecal microbiota change trend of the control and resistant groups from infection to recovery periods was more similar than that of the susceptible group.

#### 3.3.3. Differences in the Abundance of Cecal Microorganisms among the Different Groups at the Genus Level

**Figure 5:**
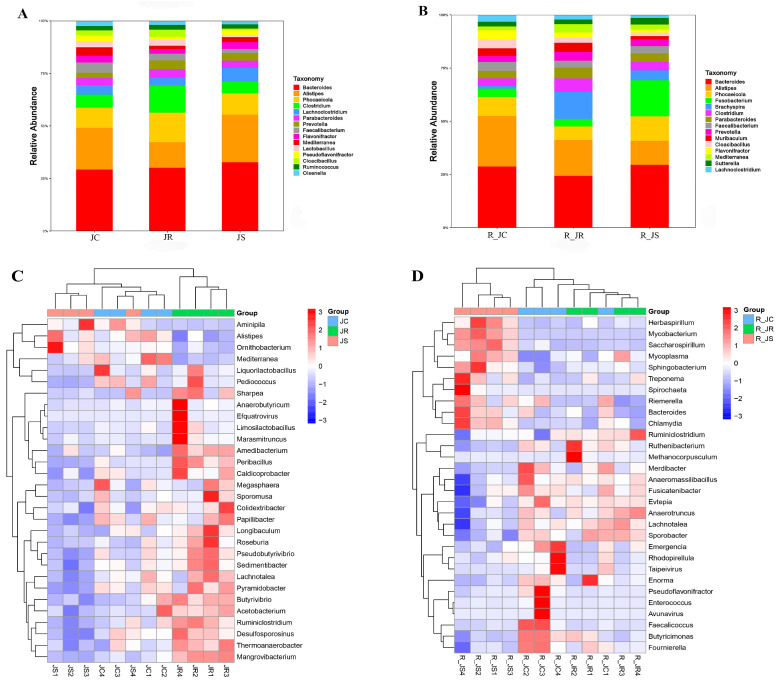
Comparative analysis of cecal microbiota at the genus level between different resistant groups during infection and recovery period. Comparison of the relative abundance of the top 15 cecal microorganisms among the JC, JR, and JS groups during the infection (**A**) and recovery period (**B**). Differential abundance analysis of cecal microbiota between different resistant groups during infection (**C**) and recovery period (**D**). Hierarchical clustering plots of the top 30 microbiota with significant differences at the genus level. The bar represents the maximum and minimum values of a set of data. Statistical analysis was performed using the Kruskal–Wallis H test (*p* < 0.05). JC, control group; JR, resistant group JS, susceptible group; R_JC, control group in the recovery period; R_JR, resistant group in the recovery period; R_JS, susceptible group in the recovery period.

The proportion of abundance in the top 15 cecal microbiota at the genus level among the different groups during the infection period and the recovery period is depicted in Figure 5A,B. During the infection period (Figure 5A), *Bacteroides*, *Alistipes*, and *Phocaeicola* were the most dominant genera of cecal microbiota, and *Alistipes* in the susceptible group had a relatively higher abundance than that in other groups. The relative abundance of *Faecalibacterium* was higher in the resistant group than in the susceptible group (3.15% vs. 1.82%), as was that of *Lactobacillus* (2.78% vs. 1.15%). During the recovery period (Figure 5B), the total microflora of *Bacteroides*, *Alistipes*, and *Phocaeicola* in the resistant group accounted for less than 50%, less than that in the other two groups.

The Scheirer–Ray–Hare test also showed that there are 90 cecal microorganisms with highly significant differences between different treatment groups (*p* < 0.01). Then the Kruskal–Wallis H test was used to identify the differential abundance of cecal microbiota between the different resistance groups. The results are shown in Figure 5C,D, and as can be seen from the clustering heatmap (Figure 5C), the resistant group was clustered into one separate category during the infection period; in addition, the relative abundance of *Roseburia*, *Butyrivibrio*, and *Ruminiclostridium* was significantly higher in the resistant group than in the other two groups, whereas the relative abundance of *Alistipes* was significantly higher in the susceptible group than in the resistant group (*p* < 0.05). Whereas, in the recovery period (Figure 5D), the susceptible group was clearly differentiated from the other two groups as seen in the clustered heatmap, and the relative abundance of *Chlamydia*, *Bacteroides*, and *Spirochaeta* in the susceptible group was significantly higher than that of the control and resistant groups (*p* < 0.05). Taken together, the changes in cecal microbiota were different in chickens with different susceptibilities to coccidia infection.

### 3.4. LEfSe-Based Analysis of the Differential Abundant Cecal Microbiota among the Different Groups after E. tenella Infection

**Figure 6:**
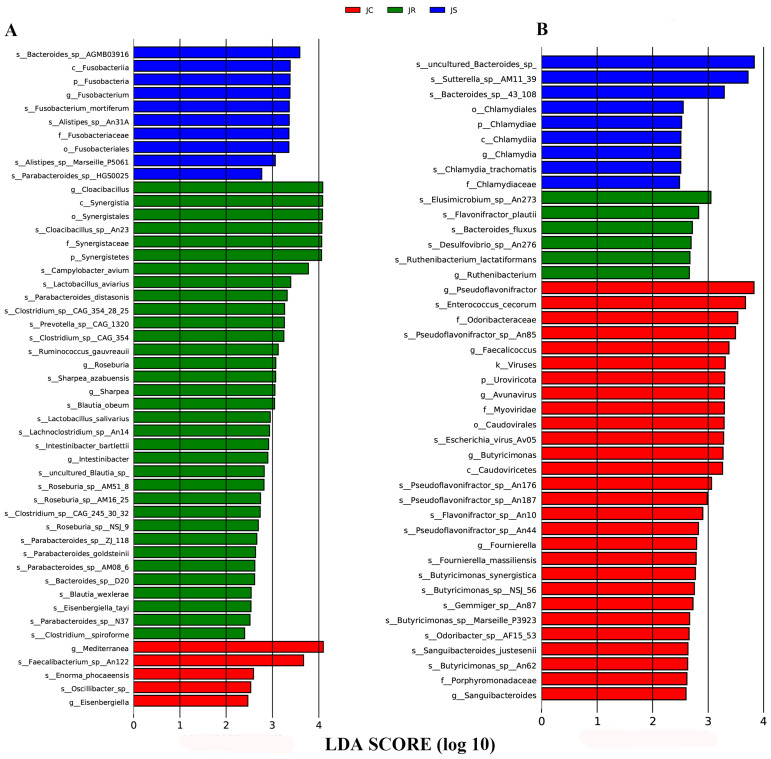
Linear discriminant analysis effect size (LEfSe)-based analysis of the cecal microbiota in the different groups. LDA scores > 2.0 and *p* < 0.05 were considered to be significant differences. Different colors indicate different groups, with red bars, green bars, and blue bars representing species that were relatively abundant in the JC, JR, and JS groups, respectively. The LDA scores for differentially abundant cecal microbiota in the different groups during the infection period (**A**) and the recovery period (**B**). JC, control group; JR, resistant group; JS, susceptible group; LDA, linear discriminant analysis.

**Figure 7:**
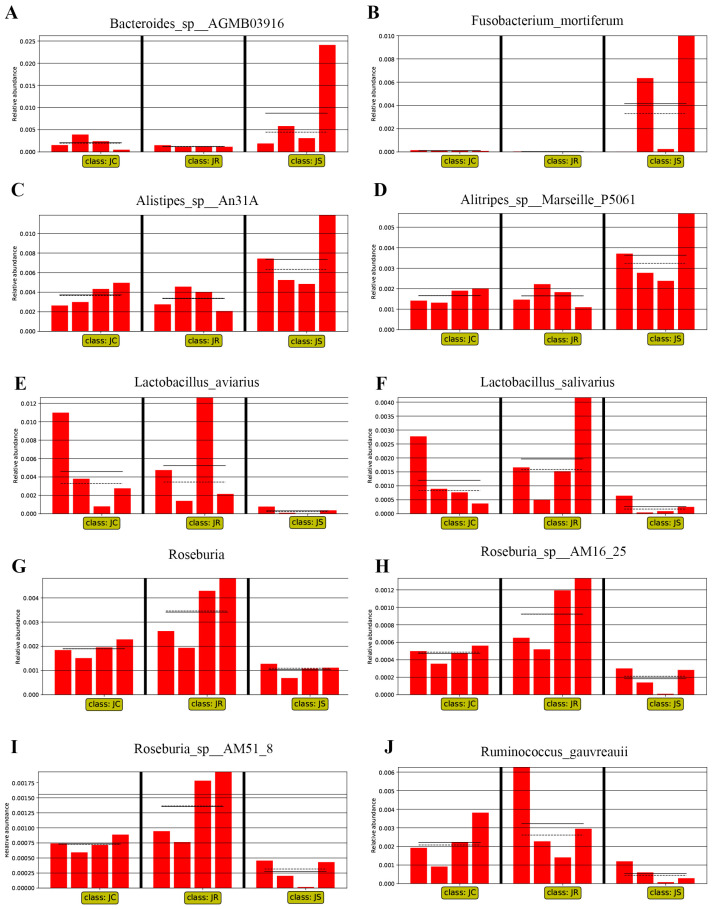
Histogram of the relative abundance of differential cecal microorganisms during the infection period; LDA scores > 2.0 and *p* < 0.05 were considered to be significant differences: (**A**) *Bacteroides_sp__AGMB03916*; (**B**) *Fusobacterium_mortiferum*; (**C**) *Alistipes_sp__An31A*; (**D**) *Alistipes_sp__Marseille_P5061*; (**E**) *Lactobacillus aviarius*; (**F**) *Lactobacillus salivarius*; (**G**) *Roseburia*; (**H**) *Roseburia_sp__AM16_25*; (**I**) *Roseburia_sp__AM51_8*; (**J**) *Ruminococcus gauvreauii*. JC, control group; JR, resistant group; JS, susceptible group. The solid lines represent the mean values of relative abundance, and the dotted lines represent the median values, and each column represents the relative abundance of each sample in each group.

**Figure 8:**
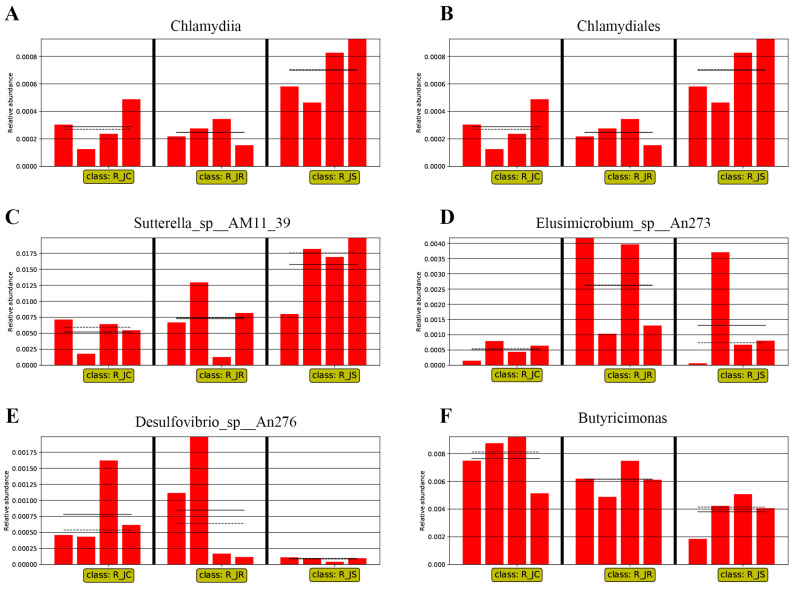
Histogram of the relative abundance of differential cecal microorganisms during the recovery period; LDA scores > 2.0 and *p* < 0.05 were considered to be significant differences: (**A**) *Chlamydiia*; (**B**) *Chlamydiales*; (**C**) *Sutterella_sp__AM11_39*; (**D**) *Elusimicrobium_sp__An273*; (**E**) *Desulfovibrio_sp__An276*; (**F**) *Butyricimonas*. R_JC, control group in the recovery period; R_JR, resistant group in the recovery period; R_JS, susceptible group in the recovery period. The solid lines represent the mean values of relative abundance, and the dotted lines represent the median values, and each column represents the relative abundance of each sample in each group.

The LEfSe algorithm was used to identify significant differences in the relative abundances of microbial taxa among the different groups during the infection period and the recovery period, which could be used as biomarkers. During the infection period, 49 biomarkers were identified using LDA scores > 2 and *p*-values < 0.05 as selection criteria (Figure 6A). Among these microbial taxa, 5 were identified for the control group, 34 for the resistant group, and 10 for the susceptible group. LDA showed that Faecalibacterium sp., Mediterranea, and Oscillibacter sp. were enriched in the control group; Roseburia, Roseburia_sp__AM16_25, Lactobacillus salivarius, Lactobacillus aviarius, and Ruminococcus gauvreauii were enriched in the resistant group; and Bacteroides_sp__AGMB03916, Fusobacterium_mortiferum, Alistipes_sp__An31A, and Alistipes_sp__Marseille_P5061 were enriched in the susceptible group (Figure 6A). During the recovery period, 43 biomarkers were identified, including 28 related to the control group, 6 to the resistant group, and 9 to the susceptible group (Figure 6B). Butyricimonas, Pseudoflavonifractor, and Enterococcus cecorum were enriched in the control group; taxa that included Ruthenibacterium, Bacteroides fluxus, and Desulfovibrio_sp__An276 were enriched in the resistant group; and the taxa Bacteroides_sp__43_108, Chlamydiia, Chlamydiales, and Sutterella_sp__AM11 39, among others, were enriched in the susceptible group (Figure 6B). Additionally, we further compared the relative abundances of some biomarkers among the different groups in both the infection and recovery periods (Figure 7 and Figure 8). During the infection period, we found that the relative abundance of Bacteroides_sp__AGMB03916, Fusobacterium_mortiferum, Alistipes_sp__An31A, and Alistipes_sp__Marseille_P5061 was higher in the susceptible group than in the control and resistant groups (Figure 7A–D). However, the relative abundance of Lactobacillus aviarius, Lactobacillus salivarius, Roseburia, Roseburia_sp__AM16_25, Roseburia_sp__AM51_8, and Ruminococcus gauvreauii was higher in the resistant group than in the other two groups (Figure 7E–J). This result indicated that these microbial taxa may be involved in fighting coccidia infection. During the recovery period, the relative abundance of Chlamydiia, Chlamydiales, and Sutterella_sp__AM11_39 increased in the susceptible group compared with that in the control and resistant groups (Figure 8A–C). Moreover, the relative abundance of Elusimicrobium_sp__An273 and Desulfovibrio_sp__An276 was higher in the resistant group than in the other two groups (Figure 8D,E).

### 3.5. Co-Occurrence Network of Cecal Microbiota

**Figure 9:**
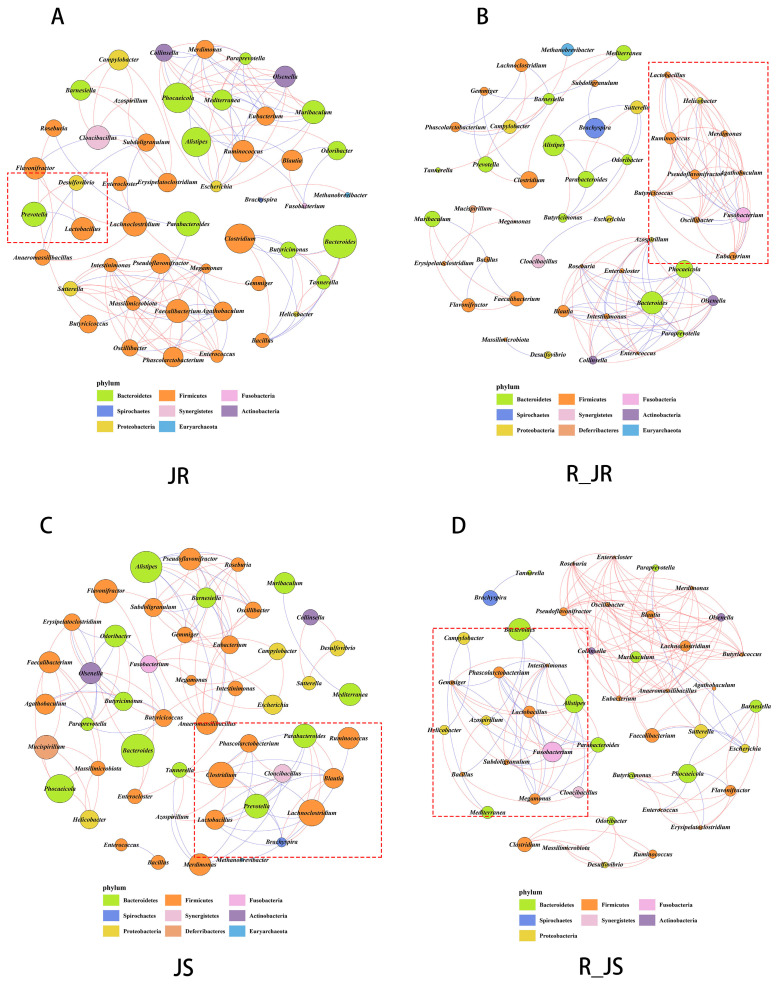
Co-occurrence network diagram of the cecal microbiota in the different groups during *E. tenella* infection and after recovery. Red edges represent positive correlations, and blue edges represent negative correlations. The colors of the circles represent different phyla of the cecal microbiota. The sizes of the nodes represent species abundance. Co-occurrence network diagram of the cecal microbiota in the JR group (**A**), the R_JR group (**B**), the JS group (**C**), and the R_JS group (**D**). JR, resistant group; R_JR, resistant group in the recovery period; JS, susceptible group; R_JS, susceptible group in the recovery period.

The co-occurrence network among the top 50 species with total abundance at the genus level is shown in Figure 9. It can be found from the co-occurrence network that the bacterial abundance of Lactobacillus, Butyricoccus, and Roseburia in both the resistance and susceptible groups in the recovery period is lower than that in the infection period. During the infection period, Lactobacillus showed significantly negative correlations with Prevotella and Desulfovibrio in the resistant group (Figure 9A). Meanwhile, during the recovery period, a significantly positive correlation was detected between Lactobacillus and Ruminococcus and Butyricoccus in the resistant group (Figure 9B). The number of symbiotic relationships between Lactobacillus and other microorganisms increased in the susceptible group during the infection period and further increased in the recovery period. During the infection period, significantly negative correlations were found between Lactobacillus and Prevotella (Figure 9C), but significantly positive correlations were found with Alistipes during the recovery period in the susceptible group (Figure 9D).

## 4. Discussion

Intestinal microbes constitute a key component of the host’s intestinal defense barrier and have a critical impact on the health and productivity of their host [21]. Coccidia infection affects the composition and integrity of the intestinal microbiota of chickens, leading to increased susceptibility to disease and posing a serious threat to the overall health and productivity of the birds [22]. Microbial diversity, evenness, and richness are vital indicators of the health of the gastrointestinal tract [23]. In this study, alpha-diversity analysis showed that the Ace index was higher in the resistant and susceptible groups than in the control group during the infection period, whereas no difference in the Shannon index was observed among the three groups. Zhou et al. [24] reported that microbial sequence diversity (Shannon index) and richness (Ace index) exhibited an increasing trend in *E. tenella*-infected chickens compared with that seen in control birds, which is consistent with our results. In contrast, a different study suggested that alpha diversity was unchanged in the intestinal microbiota of chickens following coccidia infection, even when the intestine was severely or very severely damaged [14]. In our study, during the recovery period, the Ace and Shannon indices in the susceptible group showed a decrease compared with those of the control and resistant groups. Meanwhile, Li et al. [25] found that *E. tenella* infection decreased cecal microbial diversity. According to the PCoA plots of beta diversity analysis, the results showed that the composition of the cecum microbial community among the different resistance groups exhibited significant differences between the samples during the infection or recovery period. Macdonald et al. [14] compared the beta diversity between five groups with different lesion scores after coccidia infection and found that the uninfected group had significant differences in PCoA plots compared with the group with a lower lesion level and the group with a higher lesion level, respectively. which is consistent with the results of this study. Several factors can explain this variation in results. Diet, stocking density, host genotype, age, and sex can all influence microbial composition [26,27].

The gut microbiota changes with chick age [28], and the maturing broiler chickens developed a more stable gut microbiota [29,30], and the changes are also related to the breed, geographic location, and diet of the chickens [31,32,33]. In this study, we found that *Firmicutes*, *Bacteroidetes*, and *Proteobacteria* were the most abundant phyla in the chicken cecum, and similar with Yu et al. [27]. *Firmicutes* showed a decreasing trend from infection to recovery periods in the control group, and more obviously in the infection group. For natural-growth chickens, *Firmicutes* in the cecum showed a gradual decline from week 4 to week 8 [34]. After coccidia infection, the abundance of *Firmicutes* in the infection group decreased significantly compared to the control group [27,35]. In addition, our study also found that, from infection to recovery periods, the number of microorganisms with the same trend of change between the control and resistant groups was greater than that between the control and susceptible groups. Therefore, we suggest that differences in chicken susceptibility to coccidia are responsible for differences in the cecal microbiota. In the resistant group, 25 cecal microbiota decreased significantly from the infection to recovery periods. Among them, *Coprococcus*, *Roseburia,* and *Butyricicoccus* are beneficial microorganisms and butyrate-producing bacteria [36,37,38]. Butyric acid is thought to play an important role in maintaining the health and performance of broiler chickens. Furthermore, it was shown that butyric acid could significantly restore the cecal microbiota dysbiosis caused by *E. tenella* infection in chickens [39]. In the recovery period, only five microorganisms showed significant increases, such as *Helicobacter* and *Sutterella*. *Helicobacter* is a foodborne pathogen that can colonize the intestines and livers of birds and humans, causing a variety of gastrointestinal and liver diseases [40]. In the susceptible group, 11 cecal microbiota in the infection period were significantly higher than in the recovery period, such as *Alistipes*, *Butyricicoccus*, and *Eubacterium*, which have been reported to play a role in reducing ulcerative colitis and inflammatory bowel disease, producing propionate and butyrate, and in immune regulation and inhibiting inflammation [38,41,42,43]. Studies have shown that *Alistipes* dysbiosis can be either beneficial or harmful [41]. *Alistipes* spp. was linked to several diseases, including liver fibrosis [44], colorectal cancer [45], cardiovascular disease [46], and mood disorders [47], among other potential diseases. Some studies have shown that [27,35] after *E. tenella* infection, the abundance of *Alistipes* in the cecal microbiota increased significantly, which is consistent with our results. While in the recovery period, 19 cecal microbiota significantly increased from infection to recovery periods, including *Pantoea*, *Sporomusa*, and *Pasteurella*. *Pantoea* was the first identified as the microbiota responsible for galls, wilting, soft rot, and necrosis in various plants [48] and is an opportunistic pathogen that often causes urinary tract infections and surgical wound infections [49]. *Pasteurella* is a major cause of cholera in poultry and is often responsible for significant economic losses [50]. The composition of cecum microorganisms changed after *E. tenella* infection, and the number of opportunistic pathogens increased with infection over time. Although many studies have shown that coccidian resistance is associated with host immune responses, especially intestinal immunity [13,51], further research is needed to determine whether changes in the cecal microbiota after coccidian infection affect host immune or inflammatory responses.

In this study, different resistance and susceptibility groups to coccidia showed that, during the infection period, the abundance of *Faecalibacterium*, *Lactobacillus*, *Roseburia*, *Butyrivibrio*, and *Ruminiclostridium* in the resistant group was higher than that in the susceptible group, while *Alistipes* in the susceptible group was significantly higher than that in the resistant group. In addition, to screen out potential biomarkers, we further performed LEfSe analysis on the cecal microbiota of the control, resistant, and susceptible groups during the infection period. The results also showed that *Roseburia*, *Roseburia_sp__AM16_25*, *Roseburia_sp__AM51_8*, *Lactobacillus aviarius*, *Lactobacillus salivarius*, and *Ruminococcus gauvreauii* in the resistant group, and *Bacteroides_sp__AGMB03916*, *Fusobacterium_mortiferum*, *Alistipes_sp__An31A*, and *Alistipes_sp__Marseille_P5061* in the susceptible group could be considered as a potential biomarker. Previous studies have shown that *Lactobacillus* has functions of resisting harmful bacteria colonization [52], promoting immunity in the intestinal mucosa and ameliorating intestinal tissue damage [53,54], regulating inflammatory responses [55], and relations to nutrient metabolism [56] and feed efficiency [57]. So *Lactobacillus* is an important component of the intestinal microbiota. *Faecalibacterium* has been identified as a potentially beneficial microbe for its role in glycolysis and butyrate production, which can regulate the immune system and relieve inflammation [39]. Moreover, *Lactobacillus* and *Faecalibacterium* have been demonstrated to alleviate the pathogenicity of *Eimeria* infection [53]. *Roseburia* is one of the symbiotic bacteria in the gut microbiota, which can produce SCFAs such as butyric acid, maintain intestinal immunity, and improve disease resistance [37]. *Bacteroides* can cause endogenous infections when the organism’s immune function is disordered or the microbiota is imbalanced [57]. *Fusobacteria* is a known pathogenic bacterium in the oral cavity, and some studies have shown that *Fusobacteria* and its family members are enriched in oral cancer tissues [58]. It has also been shown that hens from the same genetic line produce fewer eggs; their cecum microbes are enriched with more than 30% *Fusobacterium mortiferum*, which may be referred to as a new pathogenic bacterium [59]. Therefore, this suggests that the difference of beneficial microorganisms in different resistant groups after *E. tenella* infection may be one of the reasons for the different resistance to coccidia.

On the other hand, during the recovery period, the results showed that the abundance of *Chlamydia*, *Bacteroides*, and *Spirochaeta* was significantly higher in the susceptible group than in the control and resistant groups. Combined with LEfSe analysis, *Bacteroides_sp__43_108*, *Chlamydiia*, and *Chlamydiales* could be considered potential biomarkers in the susceptible group. *Spirochaeta* is a potential pathogen in humans and animals, including a variety of pathogenic bacteria, such as *Brachyspira hyodysenteriae*, which can cause severe hemorrhagic colitis in growing pigs [60]. *Chlamydia* can persist in its host and is associated with a variety of chronic pathological symptoms; this is the most striking feature of some pathogenic species of this bacterial genus [61]. For example, psittacosis, a zoonotic disease caused by *Chlamydia psittaci*, is associated with severe clinical manifestations in humans and may even lead to death if not promptly treated with the appropriate antibiotics [62]. In addition, in broilers, persistent infection with *Chlamydia* can lead to reduced weight gain. Despite these observations, there are relatively few studies on the clinical manifestations of chlamydial infection [63]. As a result, the susceptible group was enriched with many harmful microorganisms during the recovery period; this might be one of the reasons for the incomplete recovery and poor growth performance of the susceptible group. Combined, our results indicated that the growth and colonization of opportunistic pathogens in the intestine increases during the recovery period, leading to increased susceptibility to other pathogens, which is more apparent in susceptible chickens. Changes in the resident microbiota may be one of the factors affecting the production performance of infected chickens.

*Prevotella* is a genus of Gram-negative anaerobic bacteria [64], and it has been reported to be associated with periodontitis [65]. Additionally, studies have shown that *Lactobacillus* can suppress the excessive growth of harmful bacteria, such as *Prevotella*, in semen [66]. In this study, the co-occurrence network analyses show that, in the resistant group, *Lactobacillus* (beneficial microbiota) negatively correlated with *Prevotella* (harmful microbiota) during the infection period and positively correlated with *Butyricoccus* and *Ruminococcus* (beneficial microbiota) during the recovery period. These results suggest that the increase in *Lactobacillus* may inhibit the growth of the *Prevotella* during the infection period, whereas during the recovery period, the decrease in *Lactobacillus* led to the abundance decrease in *Butyricoccus* and *Ruminococcus* in the resistant group.

There are a huge number of indigenous chicken breeds in China; they have outstanding traits, such as coarse grain resistance, disease resistance, and good meat quality [31]. Therefore, it is of great significance to strengthen the research on local breeding chickens. Jinhai Yellow Chickens is one of the yellow-feathered meat-type breeds and has four characteristics: small body size, high meat quality, early sexual maturity, and strong stress resistance. Currently, there are few studies on the characterization of cecal microbiota with different coccidia resistance in indigenous chickens. Therefore, the results of this study could provide very important information for related research of other indigenous chicken breeds.

## 5. Conclusions

In conclusion, in the present study, we revealed the compositional differences of cecal microbiota between different resistance groups after *E. tenella* infection during the infection and the recovery period. In addition, we also revealed the potential change trend through comparative analysis of different resistance groups from infection to recovery periods. Meanwhile, we identified potential biomarkers associated with different resistance groups during the infection period and recovery period, some beneficial bacteria including *Roseburia*, *Lactobacillus aviaries*, and *Ruminococcus gauvreauii*, and some harmful bacteria such as *Bacteroides_sp__AGMB03916*, *Fusobacterium_mortiferum*, *Alistipes_sp__An31A*, and *Chlamydiia* in the resistant and susceptible groups, respectively. These results provide a reference for our understanding of the impact of coccidiosis on cecal microbiota and the differences of cecal microbial composition among different resistant hosts.

## Figures and Tables

**Table 1:** The relative weight gain in different chicken families following *E. tenella* challenge.

Family	Group	Initial Weight (g)	Final Weight (g)	Average Daily Gain (g)	Relative Weight Gain (%)
A	CG (*n* = 12)	610.80 ± 20.98	738.80 ± 39.40 ^A^	128.00 ± 20.19 ^A^	100
	IG (*n* = 20)	622.90 ± 45.70	659.10 ± 46.54 ^B^	36.20 ± 15.45 ^B^	28.28
B	CG (*n* = 12)	578.20 ± 35.32	709.20 ± 47.07 ^A^	131.00 ± 25.80 ^A^	100
	IG (*n* = 20)	585.10 ± 43.52	611.70 ± 45.57 ^B^	26.60 ± 13.32 ^B^	20.31
C	CG (*n* = 12)	594.40 ± 50.15	725.60 ± 52.32 ^A^	131.20 ± 12.99 ^A^	100
	IG (*n* = 20)	596.50 ± 31.64	618.30 ± 36.37 ^B^	21.80 ± 6.83 ^B^	16.62
D	CG (*n* = 12)	579.67 ± 12.74	704.67 ± 6.66 ^a^	125.00 ± 6.08 ^A^	100
	IG (*n* = 20)	569.50 ± 21.72	646.33 ± 28.14 ^b^	76.83 ± 16.09 ^B^	61.47
E	CG (*n* = 12)	590.60 ± 38.17	726.80 ± 38.28 ^a^	136.20 ± 11.69 ^A^	100
	IG (*n* = 20)	595.90 ± 58.62	653.40 ± 66.04 ^b^	57.50 ± 23.35 ^B^	42.22
F	CG (*n* = 12)	581.67 ± 30.81	707.00 ± 32.83	125.33 ± 29.84 ^a^	100
	IG (*n* = 20)	595.83 ± 71.12	660.17 ± 77.77	64.33 ± 16.23 ^b^	51.33
G	CG (*n* = 12)	548.33 ± 26.58	669.67 ± 27.79	121.33 ± 20.26 ^a^	100
	IG (*n* = 20)	543.00 ± 69.48	601.50 ± 84.49	58.50 ± 35.35 ^b^	48.21

CG, control group; IG, infection group. The column with different uppercase superscript letters differs extremely significantly (*p* < 0.01), while the different lowercase superscript letters differ significantly (*p* < 0.05).

**Table 2:** The coccidiosis resistance index of the seven families after *E. tenella* infection.

Family	OPG (×10^6^)	Lesion Score	ACI
A-IG (*n* = 10)	0.43 ± 0.05 ^E^	1.80 ± 1.23 ^AB^	105.28 ± 16.32 ^BC^
B-IG (*n* = 10)	0.60 ± 0.07 ^DE^	1.70 ± 1.06 ^B^	98.31 ± 12.47 ^C^
C-IG (*n* = 10)	12.42 ± 0.81 ^A^	3.00 ± 1.05 ^A^	46.62 ± 5.98 ^D^
D-IG (*n* = 10)	0.01 ± 0.01 ^E^	1.00 ± 0.00 ^B^	151.47 ± 9.18 ^A^
E-IG (*n* = 10)	1.24 ± 0.50 ^C^	1.80 ± 1.14 ^AB^	114.22 ± 9.69 ^B^
F-IG (*n* = 10)	2.64 ± 0.66 ^B^	1.83 ± 1.11 ^AB^	113.00 ± 9.65 ^B^
G-IG (*n* = 10)	1.14 ± 0.19 ^CD^	1.50 ± 1.22 ^B^	123.21 ± 19.53 ^B^

IG, infection group; OPG, oocysts per gram; ACI, anticoccidial index. The column with different uppercase superscript letters differs extremely significantly (*p* < 0.01); the same letters showed that the difference was not significant (*p* > 0.01).

**Table 3:** Comparative analysis of alpha-diversity of the three experimental groups during the infection and recovery period.

	Sample ID	Infection Period	Recovery Period
ACE	JC	2168.57 ± 144.05	1993.94 ± 320.16
	JR	2553.99 ± 123.26	2166.29 ± 374.80
	JS	2273.83 ± 174.99	1802.09 ± 511.34
Shannon	JC	4.99 ± 0.05	5.08 ± 0.27
	JR	4.79 ± 0.22	5.17 ± 0.12
	JS	4.95 ± 0.18	5.00 ± 0.53
Simpson	JC	0.97 ± 0.004	0.97 ± 0.006
	JR	0.96 ± 0.016	0.97 ± 0.003
	JS	0.97 ± 0.010	0.97 ± 0.020

JC, control group; JR, resistant group; JS, susceptible group. Significance was performed by one-way analysis of variance (ANOVA); no letter indicates insignificant difference (*p* > 0.05).

## Data Availability

The datasets presented in this study can be found in online repositories. The names of the repository/repositories and accession number(s) can be found below: NCBI; PRJNA943112.
